# A Novel Electronic Interface for Micromachined Si-Based Photomultipliers

**DOI:** 10.3390/mi9100507

**Published:** 2018-10-08

**Authors:** Leonardo Pantoli, Gianluca Barile, Alfiero Leoni, Mirco Muttillo, Vincenzo Stornelli

**Affiliations:** Department of Industrial and Information Engineering and Economics, Università degli Studi dell’Aquila, 67100 L’Aquila, Italy; gianluca.barile@graduate.univaq.it (G.B.); alfiero.leoni@graduate.univaq.it (A.L.); mirco.muttillo@graduate.univaq.it (M.M.); vincenzo.stornelli@univaq.it (V.S.)

**Keywords:** silicon photomultipliers (SiPMs), analog interfacing, second-generation voltage conveyor (VCII) interfaces, second-generation current conveyor (CCII) interfaces, integrated circuits

## Abstract

In this manuscript, the authors propose a novel interface for silicon photomultipliers based on a second-generation voltage conveyor as an active element, performing as a transimpedance amplifier. Due to the absence of internal feedback, this solution offers a static bandwidth regardless of the tunable gain level. The simulation results have shown good performances, confirming the possibility of the proposed interface being effectively used in different scenarios. A preliminary hybrid solution has also been developed using second-generation current conveyors and measurements conducted on an equivalent discrete-elements board, which is promising.

## 1. Introduction

Silicon photomultipliers (SiPMs) are becoming a highly attractive alternative to traditional photomultiplier tubes (PMTs) because they are an affordable solution, able to combine high sensitivity and detection capabilities towards low-emission phenomena, together with advantages relative to the use of integrated sensors and circuits. In addition, they have a compact and robust structure, which also makes them suitable for portable applications considering the low-power consumption of the integrated solutions. A variety of SiPMs have been developed and made commercially available in order to satisfy several applications [1,2]. Given that the SiPM is based on the use of single photon avalanche diodes (SPADs), performance can be defined and changed in terms of sensitivity, resolution, response time and driving capability [3,4,5,6]. A fast output allows, for instance, the sensor to resolve high-repetition, fast pulses. In other words, a wider active area enhances the detection capability as more SPADs detect photons identically and independently. In general, the SiPMs characteristics are mainly dependent on technology and the physical architecture of the sensor, while, on the other hand, the achievable performance of the SiPM stresses the subsequent electronic circuits that are responsible for the detection and identification of the photons.

In recent years, a great effort has been devoted to the definition of new circuital solutions for the design of suitable sensor interfaces for SiPMs. These sensors demand strict performance from the electronics, in particular those which concern the response time, the resolution, and the driving capability. This means that an agile electronic interface with a large bandwidth, low noise performance and a low input impedance is desirable in order to take advantage of the use of photomultipliers. In the literature, many solutions have already been presented [7,8,9,10,11,12,13,14]. A typical choice in particle physics design consists of the use of voltage-mode amplifiers with feedback networks, which are useful for decreasing both the input impedance and noise contribution. In general, a current-mode design approach is usually discouraged because it is useful to provide a higher speed with respect to voltage-mode solutions but also higher noise performance in experiments. Recently, a mixed-mode solution has also been presented by the same authors [15], which represented a good compromise between the achievable performances and also demonstrated a capability to be used with fast SiPMs.

In this paper, this design approach has been further investigated. It is based on a second-generation voltage conveyor (VCII) [16,17] that is able to drive very large input capacitive loads as usually happens with large SiPMs or SiPMs arrays, providing a fast response. This solution is further explored here, demonstrating even better performance and its capacity to be used for practical applications in realizing a compact integrated interface. A major point of novelty presented here, is that this voltage-current approach offers variable gain without affecting the bandwidth of the interface circuit differently from other, already published solutions.

In addition, a preliminary prototype board has also been developed with commercial components, for the purpose of testing the proposed design approach. It makes use of the AD844 operational amplifiers from Analog Devices (Norwood, MA, USA) adopted to develop current conveyors [18,19]. The unique advantage of the integrated solution, apart from the novel electronic scheme, is the absence of an internal feedback. This offers a static bandwidth regardless of the tunable gain level, together with very low voltage, and therefore, portable operation capability. This is generally true for most integrated circuit (IC) solutions accomplished by low voltage and low power battery operation and also therefore, portable capability. The Hamamatsu S13360 series SiPM characteristics have been considered for simulations and to emulate the sensors current peaks during our test sessions. The results have clearly shown that even if performance of the hybrid solution cannot be compared to those of the integrated interface simulated with a standard 0.35 um complementary metal–oxide semiconductor (CMOS) technology process from AMS Foundry, the discrete version is able to provide a good response with a reasonable delay time, given the multi-peak input signal simulating multiple-photon detection.

## 2. CMOS Integrable Solution

**Figure 1:**
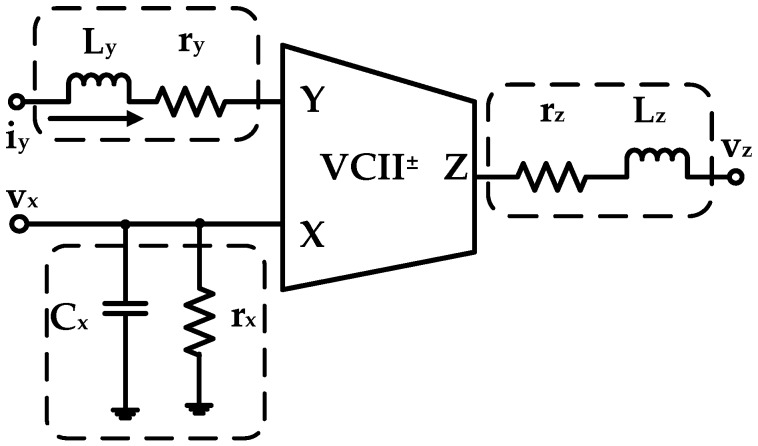
Second-generation voltage conveyor (VCII) equivalent representation. Dashed boxes highlight the parasitic components at each terminal.

The equivalent block diagram of a generic VCII is reported in Figure 1, where parasitic components have also been included and highlighted in the dashed areas. As evident, a VCII is a three-port device which exploits the dual concept of the better known second-generation current conveyor (CCII). The complete input–output relationship between each port can be extracted from the Equation (1) matrix:

(1) $$\left[\begin{array}{c} i_{x}\\ v_{y}\\ v_{z} \end{array}\right]=\left[\begin{array}{c} \frac{1}{\left(r_{x}\|1/sC_{x}\right)}\;\pm \beta \;0\\ 0\;Z_{y}\left(r_{y}+sL_{y}\right)\;0\\ \alpha \;0\;\left(r_{z}+sL_{z}\right) \end{array}\right]\cdot \left[\begin{array}{c} v_{x}\\ i_{y}\\ i_{z} \end{array}\right]$$

Analyzing Equation (1), *α* is the voltage gain between *X* input and *Z* output, which according to the CCII parallelism, should be designed as close to unity as possible. *β* is the current gain between *Y* input and *X* output and similarly to *α*, it should be designed as equal to unity. Moreover, based on the *Y*–*X* current senses, we can have a VCII^+^ (if both the currents are pointing inwards or outwards) or a VCII^−^ (if currents are pointing in opposite directions). The parameters *r_y_*, *L_y_*, *r_x_*, *C_x_*, *r_z_* and *L_z_* are the parasitic impedances related to each terminal. They should ideally be equal to zero except for *r_x_*, which should be equal to infinity. Given these considerations, we can simplify Equation (1) to Equation (2):

(2) $$I_{x}=\pm \beta I_{y},\;V_{z}=\alpha V_{x},\;V_{y}=0$$

**Figure 2:**
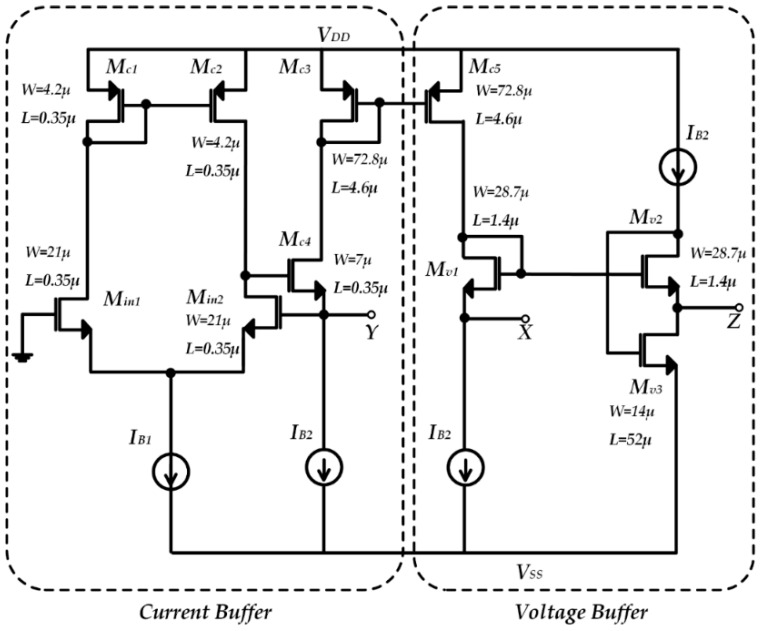
VCII transistor level implementation.

We can then conclude that the *X* terminal can be considered as a current output and hence, it should ideally have an infinite input impedance. The *Y* terminal is a current input and therefore it should be designed with zero input impedance, similarly to the *Z* terminal, which on the other hand can be considered as a voltage output. The main VCII^+^ building block used in the SiPM interface is shown in Figure 2. The transistor dimensions are reported in the same schematic. Its design was created using a standard Austria micro systems (AMS) 350 nm CMOS technology with a supply voltage of ±1.65 V. As highlighted, it consists of a current buffer and a voltage buffer. In particular, *M_c_*_4_, *M_in_*_1,2_, and *M_c_*_1,2_ employ a gain boosted common gate amplifier, which together with the current mirror *M_c_*_3,4_, conveys the *Y* input current to the *X* node, implementing the *I_x_* = *βI_y_* relationship. On the other hand, *M_v_*_2,3_ forms a flipped-voltage-follower buffer, mirroring the *X* input voltage (suitably shifted by M_v1_) to the *Z* node (*V_z_ = αV_x_*).

**Figure 3:**
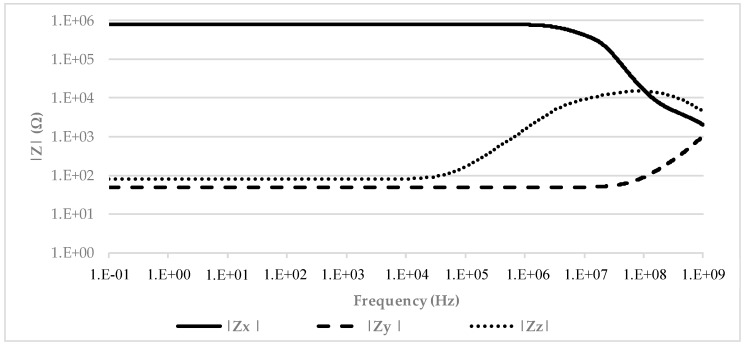
VCII impedances vs. frequency behavior.

**Figure 4:**
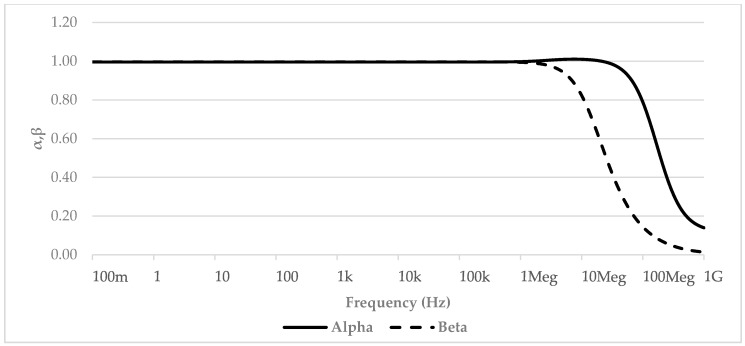
VCII *α* and *β* vs. frequency behavior.

Figure 3 shows the simulated terminal impedances. As evident, the *X*, *Y*, and *Z* nodes demonstrated a resistive behavior with a wide bandwidth and a value of 800 kΩ, 49 Ω, and 79 Ω, respectively. Figure 4 shows the *α* and *β* parameter trends in the frequency domain. Again, we can see an almost unitary value for both of them with a bandwidth greater than 100 MHz and 10 MHz, respectively.

**Figure 5:**
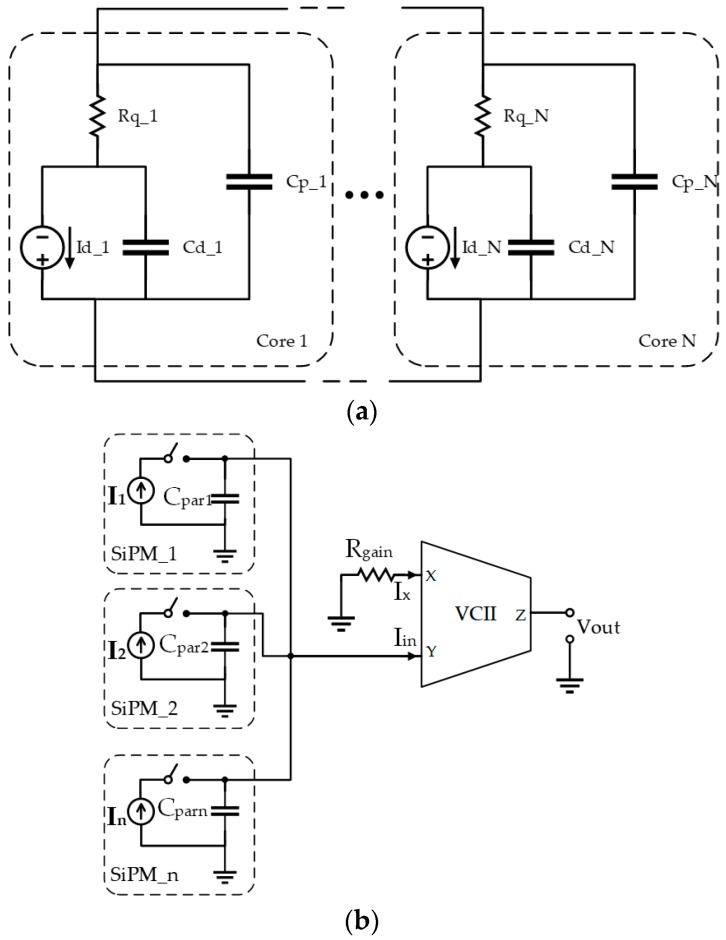
(**a**) Silicon photomultipliers (SiPM) equivalent model; (**b**) the proposed VCII-based SiPM array interface where C_par_i_ = Σ C_p_N_.

The actual SiPM interface is shown in Figure 5. As mentioned in the introduction, we considered the equivalent electrical characteristics of the Hamamatsu S13360 series SiPM (Hamamatsu Photonics, Hamamatsu, Japan). They are multi-pixel photon counters, specially made for precision measurements such as flow cytometry, DNA sequencing, laser microscopy, and fluorescence measurements. The Hamamatsu S13360-3025CS was used as a reference for our design. It has 14,400 pixels and an effective photosensitive area of 3.0 mm × 3.0 mm, with an equivalent parasitic capacitance of 320 pF. For bandwidth performance evaluation, we measured the capacitance range of the Hamamatsu S13360 family, which was 60–1280 pF. All of these parameters contributed to the definition of the equivalent input signal used for both the simulations and measurements. From the circuit front-end point of view, one of the most important SiPM parameters was the equivalent total parasitic capacitance, as observed at the output terminals of the photomultiplier array. This affects the bandwidth, and thus the time response of the front-end circuit. Particular attention must therefore be paid to the design of the input stage. In addition, because the considered current peaks are quite low in amplitude, the circuit should be designed so as to have a very low noise feature. The equivalent model of a single SiPM which has suitable regard for these design constraints is shown in Figure 5a. As can be seen, the core of the multiplier is composed of a current source and a ‘diode capacitance’ emulating the behavior of the single photon avalanche diode (SPAD). To allow the device to shut down after an event, a quenching resistor was added in series with the SPAD. Finally, a parallel capacitor C_p_N_ was placed to account for the total single-core parasitics. The SiPM was then obtained as an array of N repetitions of this basic structure. A switch (see Figure 5b) was also added in series with the SiPM in order to be able to decide the exact time of an occurrence. The actual interface is shown in Figure 5b. It consists of a single VCII performing as a transimpedance amplifier (TIA). The photomultiplier (or array of photomultipliers) output is connected to the *Y* terminal. By analyzing the *X* terminal and using the first relationship of Equation (1), we can write:

(3) $$V_{x}=I_{x}R_{gain}\approx \pm \beta I_{in}R_{gain}$$

Knowing that *V_z_* = *αV_x_* = *V_out_* we can conclude that:

(4) $$V_{out}=\alpha V_{x}\approx \pm \alpha \beta R_{gain}I_{in}\approx R_{gain}I_{in}$$

**Figure 6:**
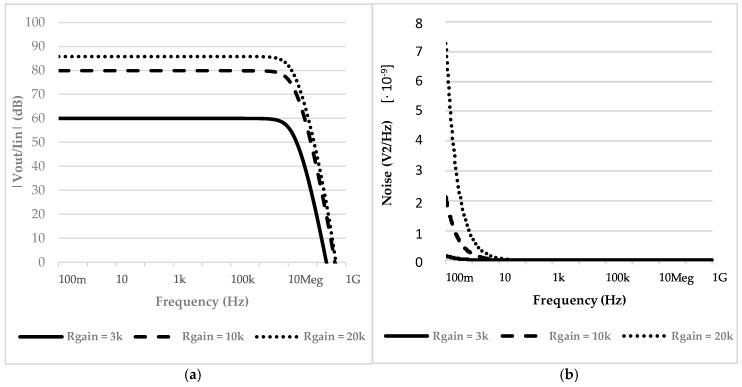
(**a**) Transimpedance amplifier (TIA) transfer function at different gain levels; (**b**) TIA output equivalent noise at different noise levels.

The main results of the simulation conducted on the transimpedance amplifier are reported in Figure 6. Figure 6a shows the transfer function of the amplifier, which confirms Equation (4). By varying the gain resistor it is possible to achieve a transimpedance gain of up to 90 dB while keeping noise levels almost constant, as shown in Figure 6b.

The gain limitation resides in the value of the gain resistor. It has to remain well below the VCII X node input resistance in order for Equation (4) to be valid. A remarkable behavior of the VCII that we present is the fact that increasing the gain does not affect the output bandwidth. This is because the transfer function zero crossing frequency varies according to the gain level.

**Figure 7:**
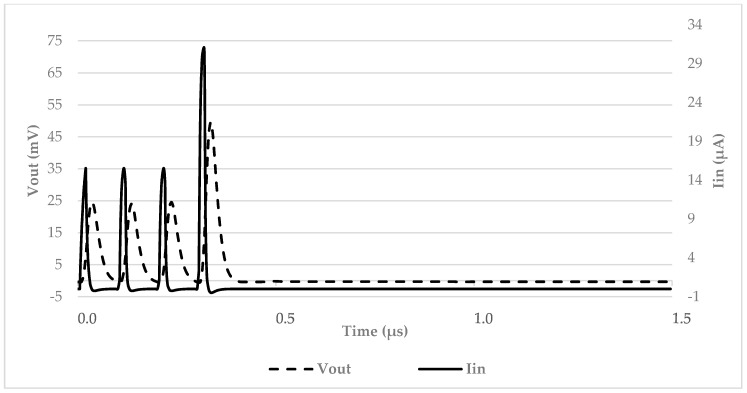
Time domain response of the interface to a train of SiPM current pulses at different amplitude levels.

The response of the interface to the SiPM output was obtained by emulating the photomultiplier current pulse in response to one or more photons hitting its surface. The results are shown in Figure 7. As can be seen, the TIA can detect a short series of pulses effectively converting them into voltage pulses. From the same figure it is also clear that the interfacing circuit is able to detect situations where multiple photons hit a SiPM or when different photons hit different SiPMs at the same time in a SiPM array, without saturating its output (i.e., while still being capable of ‘counting’ the number of photons that reached the sensors).

**Figure 8:**
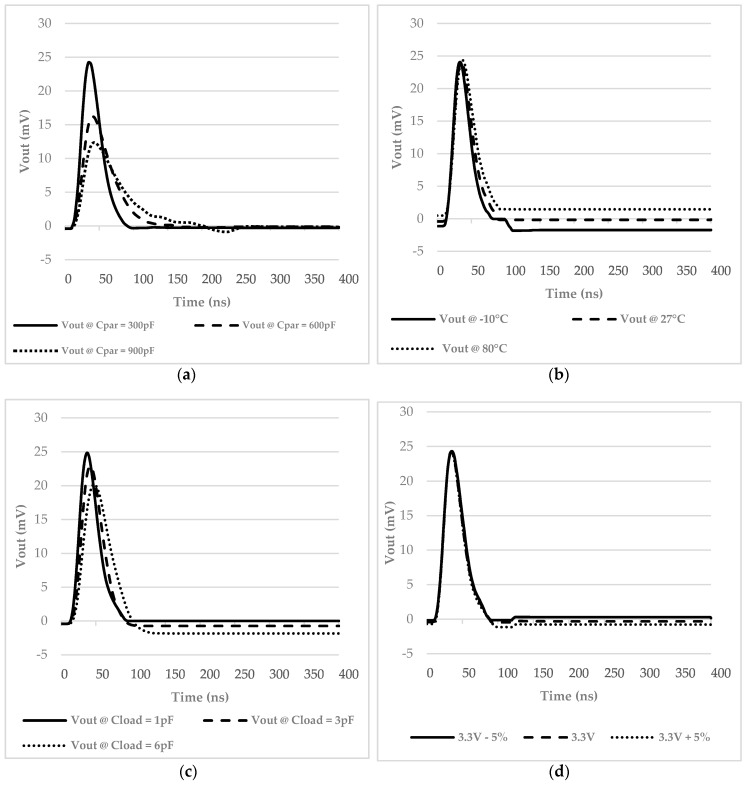
(**a**) Time domain output voltage for different parasitic capacitances, simulating the interface to be used with an array of SiPMs; (**b**) interface output voltage variations at different temperatures; (**c**) interface output voltage for three different capacitive loads connected to the VCII Z node; (**d**) interface output voltage for ±5% supply voltage variations.

Figure 8 shows an ensemble of different working conditions. Figure 8a confirms the feasibility for the interface to be used with an array of photomultipliers, as its output voltage is not critically distorted by variations in the SiPMs parasitic capacitance. Figure 8b shows the temperature variations of the interface output voltage, whereby differences from −10 °C to 80 °C are negligible, making the interface suitable to work in different environments. Figure 8c shows the same TIA output magnitude at different capacitive load levels; from 1 pF to 6 pF. Again, we can see minimal differences, meaning that the interface output stage is capable of driving further processing stages, as well as being connected directly to a chip output pad. Figure 8d shows that the interface output does not vary for a ±5% supply voltage variation, reinforcing what was previously stated about the versatility of our proposal. Finally, statistical (corner) simulations considering the utilized CMOS technology parameters were also performed, showing a 10% variation in the performances in terms of amplitude reproductivity, confirming the feasibility of the proposed solution.

## 3. Hybrid Solution, Simulations and Test

**Figure 9:**
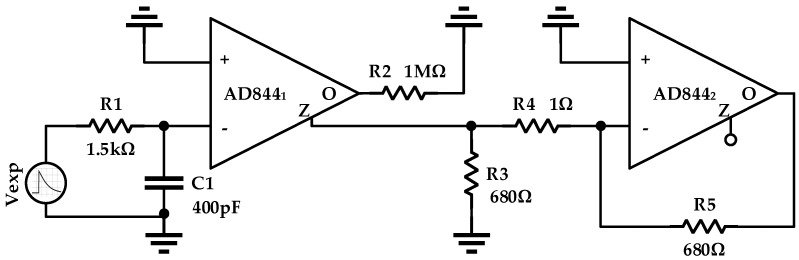
Simplified schematic of the discrete interface.

Since the VCII is not available as a commercial component, the proposed design approach has been tested with a preliminary hybrid prototype by using high speed monolithic operational amplifiers (OP-AMPs)—the AD844 from Analog Device (Analog Devices, Inc., Norwood, MA, USA), and implementing a voltage buffer and current buffer. In References [18,19] it has been demonstrated that this OP-AMP can be successfully applied to create a CCII in practical applications and so it can be adopted to obtain a transimpedance gain as in the integrated solution, already proposed in Section 2. In Figure 9, a simplified schematic of the discrete interface is reported. Two AD844 were used. The first one is devoted to the current-to-voltage conversion, while the second one is used as a traditional operational amplifier, taking advantage of the high slew rate provided by this component.

The SiPM source is simulated with a self-defined exponential current source with a very large parallel capacitor simulating the output capacitive load provided by the sensors. The output of the first stage is received on the output current terminal of the AD844 and it is connected to the input stage of the following OP-AMP, which can be used to create an inverting or non-inverting gain stage without significantly affecting the overall performance.

**Figure 10:**
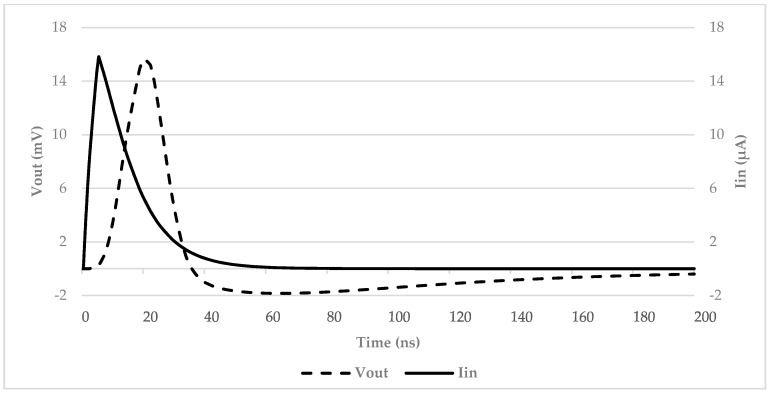
Simulation results on the prototype board: input current and output voltage of the discrete interface.

In order to investigate the performance that can be achieved with this simple architecture, a current pulse with an amplitude of 16 μA and a duration up to the minimum value of 30 ns was used as the input source. Figure 10 clearly shows that the proposed solution, even considering fast SiPM signals, is able to preserve almost the same shape factor of the input current pulse, with a minimum delay of about 15 ns. This is reasonable in our opinion considering the technological limits of a discrete prototype.

**Figure 11:**
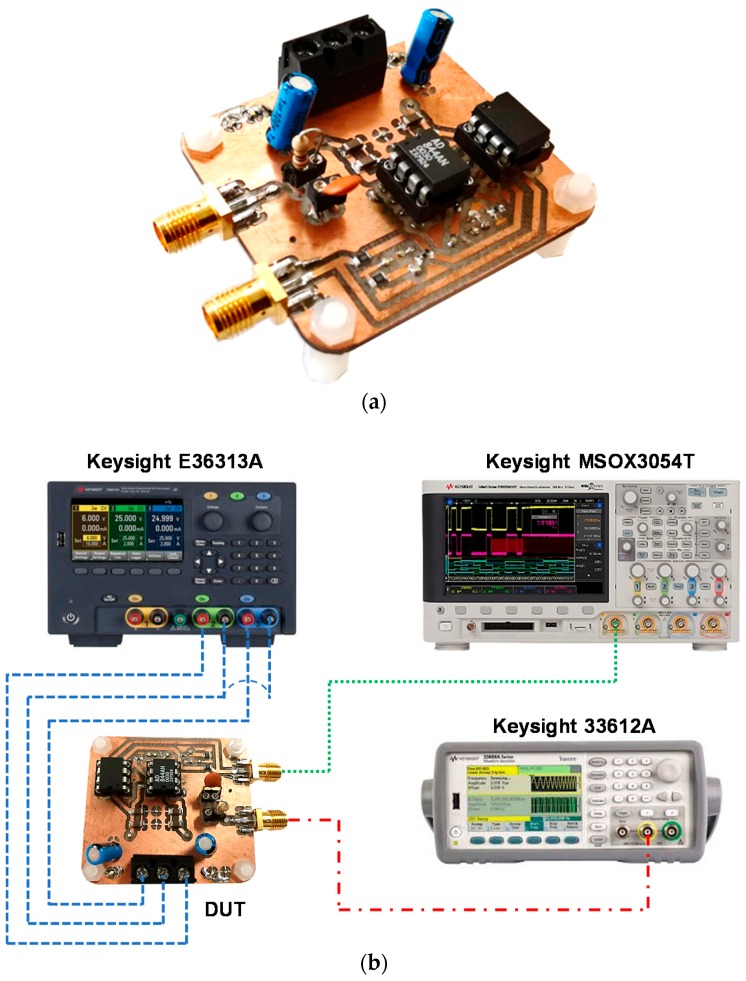

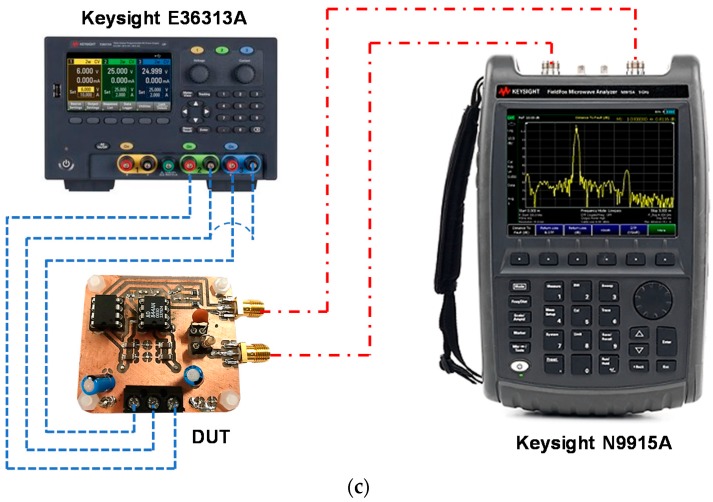
(**a**) Final prototype board; test benches: (**b**) for time domain measurements; (**c**) for frequency domain measurements.

**Figure 12:**
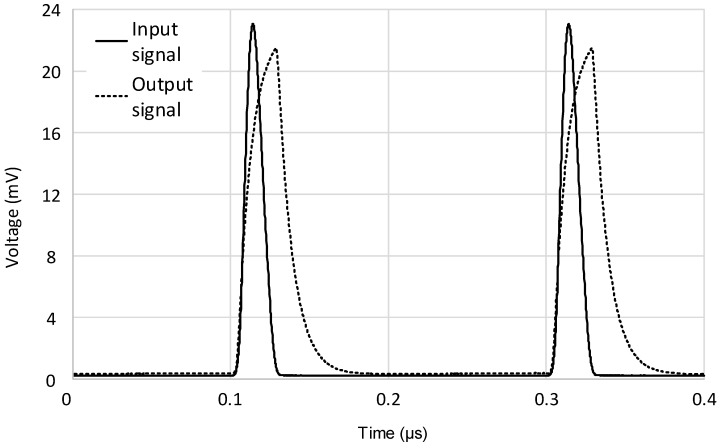
Multi-pulse input signal (continuous line) and measured output response (dotted line) of the discrete interface.

**Figure 13:**
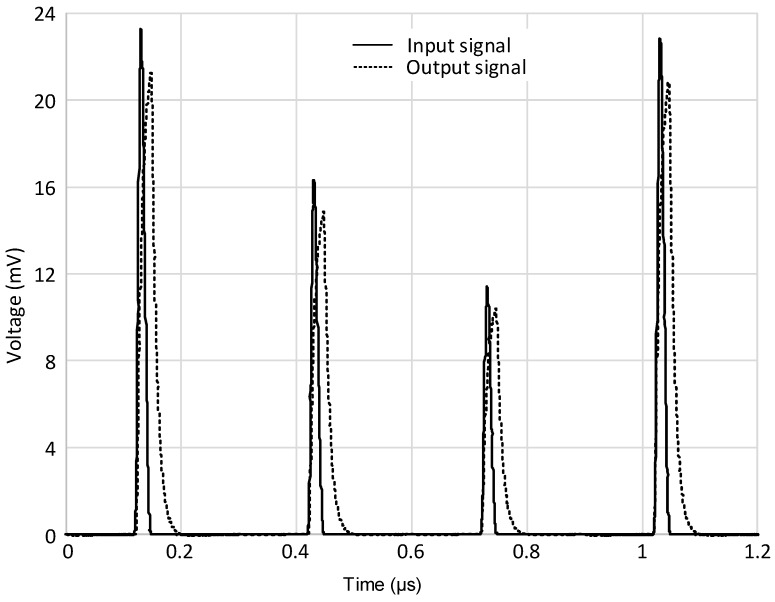
Multi-pulse variable input signal (continuous line) and measured output response (dotted line) of the discrete interface.

**Figure 14:**
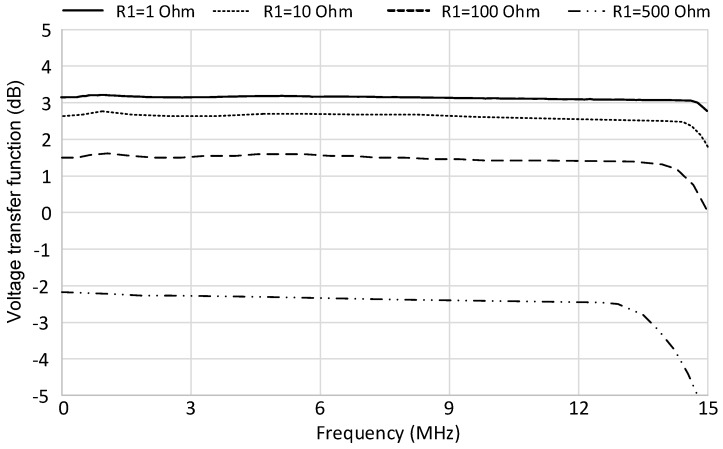
Measured voltage transfer function for different values of the input resistance R1.

A prototype board was also fabricated on a low-losses perfboard FR4 substrate with our facilities. In Figure 11a, the final hybrid prototype board is presented. The input signal was generated with the Keysight 33600A (Keysight Technologies, Santa Rosa, CA, USA) signal generator in order to have a fully characterized input source, which is useful to analyze the circuit behavior. The board has been tested in several conditions and both time and frequency domain measurements have been performed. Examples of the test bench are shown in Figure 11b,c. In the time domain, a single current pulse with different amplitudes and durations, followed by a multi-pulse signal of both fixed and variable amplitudes, was considered in order to evaluate the circuit performance with respect to an agile, time-varying incoming signal. A couple of examples are reported in Figure 12 and Figure 13. In detail, Figure 12 shows the measured output signal of the described interface when a periodic input signal is applied, consisting of a pulse train with fixed amplitude and duration while in Figure 13, the input pulse train shows variable amplitude. It is important to notice that in both cases the designed interface is able to track any changes of amplitude or repetition time with a small delay time but without significantly affecting the shape of the output pulse, even when considering very short input pulses with a minimum duration of 30 ns. Some results in the frequency domain are also reported in Figure 14. The AC voltage transfer function of the proposed circuit was measured with the Keysight N9915A FieldFox Microwave Analyzer (Keysight Technologies, Santa Rosa, CA, USA). The measured results demonstrate the very large bandwidth that can be achieved with the proposed solution. The analysis was carried out for different values of the input resistance R1, and the results are congruent with the expected behavior. The voltage gain decreased for the largest values of R1, meaning that the transimpedance gain increased accordingly, as expected from Equation (4).

## 4. Conclusions

In this manuscript a novel interface for SiPMs is proposed and addressed with circuitry details. The proposed solution used a transimpedance amplifier in order to convert the incoming current pulses from the SiPMs into corresponding voltage pulses by means of a VCII. This design approach has shown promising results, offering a compact and integrated solution in CMOS technology. A preliminary hybrid solution was also developed and evaluated using AD844 components (Analog Devices), OP-AMPs suitable for implementing CCIIs. The results have demonstrated the feasibility of the proposed solution.
